# Whole-Genome Deep Sequencing Reveals Host-Driven *in-planta* Evolution of Columnea Latent Viroid (CLVd) Quasi-Species Populations

**DOI:** 10.3390/ijms21093262

**Published:** 2020-05-05

**Authors:** Parichate Tangkanchanapas, Annelies Haegeman, Tom Ruttink, Monica Höfte, Kris De Jonghe

**Affiliations:** 1 Plant Sciences Unit, Flanders Research Institute for Agriculture, Fisheries and Food (ILVO), Burgemeester Van Gansberghelaan 96, 9820 Merelbeke, Belgium; parichate.tangkanchanapas@ilvo.vlaanderen.be (P.T.); annelies.haegeman@ilvo.vlaanderen.be (A.H.); tom.ruttink@ilvo.vlaanderen.be (T.R.); 2 Department of Plants and Crops, Faculty of Bioscience Engineering, Ghent University, Coupure links 653, 9000 Ghent, Belgium; monica.hofte@ugent.be

**Keywords:** Columnea latent viroid, population study, quasi-species, mutations, viroid secondary structure, amplicon sequencing, viroid–host interaction, high-throughput sequencing, variant analysis

## Abstract

Columnea latent viroid (CLVd) is one of the most serious tomato diseases. In general, viroids have high mutation rates. This generates a population of variants (so-called quasi-species) that co-exist in their host and exhibit a huge level of genetic diversity. To study the population of CLVd in individual host plants, we used amplicon sequencing using specific CLVd primers linked with a sample-specific index sequence to amplify libraries. An infectious clone of a CLVd isolate Chaipayon-1 was inoculated on different solanaceous host plants. Six replicates of the amplicon sequencing results showed very high reproducibility. On average, we obtained 133,449 CLVd reads per PCR-replicate and 79 to 561 viroid sequence variants, depending on the plant species. We identified 19 major variants (>1.0% mean relative abundance) in which a total of 16 single-nucleotide polymorphisms (SNPs) and two single nucleotide insertions were observed. All major variants contained a combination of 4 to 6 SNPs. Secondary structure prediction clustered all major variants into a tomato/bolo maka group with four loops (I, II, IV and V), and a chili pepper group with four loops (I, III, IV and V) at the terminal right domain, compared to the CLVd Chaipayon-1 which consists of five loops (I, II, III, IV and V).

## 1. Introduction

*Columnea latent viroid* (CLVd), a member of the *Pospiviroid* genus, is one of the smallest plant pathogens, consisting of a non-coding, naked, circular single-stranded RNA molecule of 367 to 374 nucleotides [1,2,3]. Like other pospiviroids, the genome of CLVd naturally forms a rod-like secondary structure with incomplete self-complementary base-paring within the RNA molecule [4,5]. Five domains can be distinguished in this secondary structure: the terminal left (TL), pathogenic (P), central (C), variable (V) and terminal right (TR) domain. CLVd was first found in symptomless ornamental lipstick vine (*Columnea erythrophea*) in the state of Maryland, USA [6]. CLVd has a limited host range and mainly affects crops such as tomato (*Solanum lycopersicum*), potato (*Solanum tuberosum*), eggplant (*Solanum melongena*), chili pepper (*Capsicum annuum*), petunia (*Petunia*×*hybrida*), bolo maka (*Solanum stramoniifolium*), Gynura (*Gynura aurantica*), edible chrysanthemum (*Glebionis coronaria*) and cucumber (*Cucumis sativus*) [6,7,8,9,10]. Seed transmission of CLVd has been shown in tomato, petunia, cucumber and pepper [7,9,11,12,13]. In Thailand, CLVd was first discovered in an open-field tomato seed production facility in the Khon Kaen province in 2004 [14]. This was caused by CLVd contamination of imported tomato seeds that were then post-entry quarantine regulated and eradicated shortly afterwards [8,15]. However, CLVd was introduced again by the same cause, and spread in 2013 [8]. At present, specifically in Thailand as well as other regions, CLVd is causing one of the most serious tomato diseases, leading to substantial economic losses by affecting productivity, fruit quality and international seed trade [11,16]. CLVd-Chaipayon-1 (GenBank Accession No. KM214216) was first found in a tomato hybrid-seed production site in the northeastern region of Thailand in 2014. This CLVd isolate causes intermediate symptoms in tomato, bolo maka and Thai round eggplant compared to most of the currently known Asian isolates [8].

Viroids of the *Avsunviroidae* family are replicated by a nuclear-encoded polymerase (NEP) localized in chloroplasts, while members of the *Pospiviroidae* family are replicated by DNA-dependent RNA polymerase II (Pol II) in their plant host nucleus [17]. When these host polymerases start to use RNA instead of regular DNA as the template, the mutation rate increases. High mutation rates during viroid replication (e.g., 2.5 × 10^−3^ per round of replication for *Avsunviroidae* [18], 5 × 10^−3^ for *Pospiviroidae* [19] and 1.43–2.63 × 10^−4^ determined based on full-length potato spindle tuber viroid (PSTVd) deep sequencing [20]) lead to viroid populations containing a mixture of sequence variants, also denoted by the concept of quasi-species [19,21,22]. Repeated replication of a viroid founder sequence, with mutations accumulating in subsequent progenitor replicates, generates swarms (or ”clouds”) of highly similar but not identical viroid variants, each with varying degree of fitness in a given host or environmental condition [21,22,23,24]. Over time, natural selection (i.e., infection mechanism, cell to cell and long-distance intracellular movement, avoidance of host plant defense mechanism such as RNA silencing) results in in planta evolution of the quasi-species [21]. The dominant viroid genome sequences within the population gradually changes, thus adapting viroids to their hosts. The existence of quasi-species has been reported for *Potato spindle tuber viroid* (PSTVd) [19,25,26,27], *Citrus exocortis viroid* (CEVd) [28,29,30,31,32], *Chrysanthemum stunt viroid* (CSVd) [33], *Tomato chlorotic dwarf viroid* (TCDVd) [26], *Coleus blumei viroid* (CbVd) [34], *Hop stunt viroid* (HSVd) [35,36], *Citrus bent leaf viroid* (CBLVd) [37], all members of the *Pospiviroidae* family, and *Peach latent mosaic viroid* (PLMVd), belonging to the *Avsunviroidae* [38,39]

In this work, a highly reproducible and sensitive quasi-species genomic fingerprinting method based on indexed amplicon sequencing was implemented and validated. The method makes it possible to directly obtain full-length genome sequences of all the CLVd variants, while maintaining their relative frequency in the population. Unlike other methods such as total or small RNA sequencing [19,40,41,42], sequence assembly or reconstruction is not needed. The methodology allowed to study CLVd quasi-species population evolution, define the mutation profiles and the effects of mutations on the viroid secondary structure, host specificity and evolutionary selection of single-nucleotide polymorphisms (SNP) pairs in a number of solanaceous host plants. Following initial inoculation of an infectious dsDNA CLVd-Chaipayon-1 isolate on tomato cv. Insaf, several host species including chili pepper, Thai round eggplant, bolo maka and two cultivars of tomato were subsequently mechanically inoculated, and the evolution of the viroid variant spectrum was monitored using whole-genome deep amplicon sequencing.

Our study focuses on the evolution of distinct CLVd populations across a range of host species, and the finding that the quasi-species sequence spectrum consistently depends on the host species. Furthermore, we analyzed the effect of the hotspot mutation sites on the stability of the secondary structures of CLVd-variants.

## 2. Results

### 2.1. Infectivity of a dsDNA CLVd-Chaipayon-1 Founder Isolate

As initial inoculum for CLVd infection, dsDNA of the CLVd isolate Chaipayon-1 (GenBank accession KM214216) and seven other isolates (Accessions DQ061193, DQ061192, JF742632, JF742633, KM214222, JF446916 and JF446934) was prepared. PCR analysis with CLVd specific primers showed that the initial inoculum of the eight clones consisted of self-circularized and multimeric forms (Figure S1). Tomato cultivar Insaf, bolo maka, chili pepper and Thai round eggplant were inoculated with dsDNA CLVd of the eight clones. The infectivity rate across different plant replicates of the selected host species was very low (Table S1). Only one tomato plant (cv. Insaf) inoculated with the Chaipayon-1 isolate and one tomato plant (cv. Insaf) inoculated with the Niruj-18 isolate (Accession KM214222) showed evidence of a systemic infection with the viroid RNA (Figure S2), which was confirmed by reverse transcription polymerase chain reaction (RT-PCR). Both plants also showed the typical CLVd disease symptoms. In a next step, because the Niruj-18 infected plant died, the tomato plant (Insaf) infected with the Chaipayon-1 isolate was used to inoculate five hosts, including two tomato cultivars (Insaf and Rutgers), bolo maka, chili pepper and Thai round eggplant. After inoculation with the secondary inoculum, all plants showed the specific viroid symptoms within 3 to 6 weeks depending on the plant species, and the presence of the viroid was confirmed using RT-PCR (data not shown).

### 2.2. Whole-Genome Deep Amplicon Sequencing of Quasi-Species Populations

**Figure 1:**
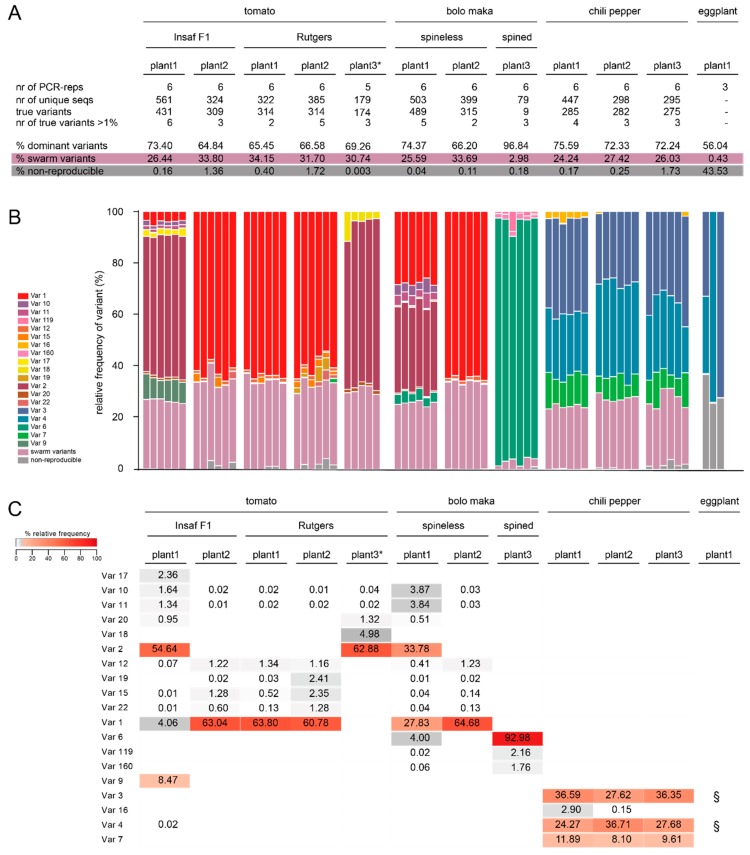
Columnea latent viroid (CLVd) variant spectra in tomato cultivars Insaf and Rutgers, bolo maka, chili pepper and Thai round eggplant. (**A**) Summary statistics of the number of variants per plant. Reproducibility across PCR replicates separates false variants (non-reproducible) from true variants (reproducible in all PCR replicates). Number of dominant variants (>1% relative frequency) per plant. Variant abundance is expressed as % relative frequency based on read depth per sample. (**B**) Relative frequency per variant per PCR replicate. The class labeled swarm variants contains all true variants (present in all PCR replicates per plant) with low frequency in the quasi-species population (<1% frequency per plant). The class labeled non-reproducible contains all false variants (reads derived from variants observed in less than 0.01% of at least one of the PCR replicates per plant). The order of plants is the same as in panel A) and all individual PCR replicates are shown per plant. (**C**) Relative frequency per dominant variant per plant. Values are average variant frequencies across PCR replicates per plant. Bi-colored heat map displays variants in the frequency range 0–5% as white-to-grey false color scale, and variants in the frequency range 5–100% as shades of red. § indicates the variants (Variant3 and Variant4) are present in these samples, but at non-reproducible variant frequencies. * indicates that for Rutgers plant3 all values are calculated across 5 PCR replicates.

In order to analyze the quasi-species sequence variation among the CLVd viroids amplified via RT-PCR, we adapted the PCR step slightly by incorporating sample-specific barcodes into the PCR primers, which allowed pooling all PCR products before Illumina adapter ligation. Using massive parallel Illumina amplicon sequencing, we then identified all full-length sequence variants per quasi-species population. Throughout this text, the term “variant” refers to one complete viroid genome sequence (except for the primer sequences) in the quasi-species population. Here, we validated the procedure by grouping all sequences per unique sequence variant, calculating their relative frequency per population and comparing variant identity and frequency between PCR replicates (technical reproducibility), plant replicates (biological reproducibility) and finally host species (quasi-species adaptation). We obtained on average 133,449 CLVd reads per PCR replicate. A high number of unique CLVd sequences were found in each PCR replicate (Figure 1A). The total number of unique sequences was consistent across independent PCR replicates of the same plant and varied from an average of 79 unique sequences (bolo maka plant 3) to 561 unique sequences (tomato plant 1 cv. Insaf). However, the original CLVd Chaipayon-1 sequence was not found in any host plant. Rarefaction analysis (Figure S3) showed that in most cases about 10,000 reads are enough to reach saturation in sequence complexity per PCR replicate, except for tomato plant 1 (cv. Insaf) in which a plateau was only reached at about 70,000 to 100,000 reads, suggesting that the quasi-species population contains a fraction of low frequency variants that can only be detected by ultra-deep sequencing.

### 2.3. Identification of True Variants and Analysis of Variant Frequency Distribution

#### 2.3.1. Reproducibility and Quasi-Species Composition

Next we checked the consistency of detection of variants across the PCR replicates per sample in order to discriminate between unique sequences derived from ”true” variants (expected to occur in all PCR-replicates per plant) versus unique sequences derived from sequencing errors (expected to occur in only one or few PCR replicates per plant, but absent in the other replicates). The vast majority of the sequence variants were observed in all available PCR replicates per plant sample. A minor fraction of the unique sequences was only observed in few PCR replicates per plant, suggesting technical noise such as PCR amplification artefacts and base calling errors (Figure 1A). Next, we focused on true variants by only retaining sequence variants that were observed in all PCR replicates per plant. The average relative frequency across all PCR replicates was calculated, and we examined the variant frequency distribution of the quasi-species that evolved in each independent plant (Figure 1B). On average, only around 0.5% of the total reads per PCR replicate was taken by non-reproducible sequences (Figure 1A), except in the non-reproducible eggplant samples. These analyses showed that viroid quasi-species typically consist of two to six “major variants” that together make up about 65*–*75% of the population, while about 25*–*35% of the reads consist of a few hundred low frequency variants (<<1%), collectively called “swarm variants” below.

Our data were derived from true variants (reproducible across six independent PCR replicates) and show that whole-genome viroid quasi-species sequencing is sensitive for low frequency variants (at least as low as 0.025% as shown in bolo maka plant 1). In contrast, the frequency spectrum of non-reproducible unique sequences (false variants, likely derived from randomly distributed sequencing errors) was strongly skewed towards extremely low frequencies, usually below 0.01*–*0.005%.

#### 2.3.2. Variant Distribution

Across all host species tested, we identified 19 major variants with a frequency of at least 1% in at least one plant. Next, we compared major variant spectra of plants within species and between species. A varying degree of consistency in variant spectra of replicate plants within a given host species was observed (Figure 1B,C). For instance, all three chili pepper plants showed almost identical sequence variant spectra, dominated by three variants (Variant3, Variant4 and Variant7), with consistent relative variant frequency (on average 33.5%, 29.6% and 9.9%, respectively). In tomato cultivars Insaf and Rutgers, either Variant1 or Variant2 were consistently the most prevailing variants, with clear dominance of one single variant at around 55*–*65% frequency in the quasi-species population. Insaf plant 1 contained the dominant Variant2 (54.7%) combined with a few more major variants (Variant9, 8.5%; Variant1, 4.1%; Variant17, 2.4%, Variant10, 1.6%; Variant11, 1.3%) in addition to 425 low frequency swarm variants. In contrast, Variant1 was dominant in spineless bolo maka plant 2 (64.7%) combined with Variant12 (1.3%) and 313 low frequency swarm variants, while spineless bolo maka plant 1 displayed a mixture of Variant1 (27.8%), Variant2 (33.8%) and a few more minor variants (Variant6, Variant10 and Variant11, all around 4%), together with 484 low frequency swarm variants. Spined bolo maka plant 3 contained a single dominant Variant6 (93.0%) but showed no low frequency swarm variants. Additionally, the CLVd population in the single Thai round eggplant could not be amplified, as evidenced by low PCR amplicon yield (data not shown), and non-reproducible PCR replicates (Figure 1B). The major variants detected were Variant3, Variant4 and Variant14 in varying relative frequencies across three PCR replicates, while no low frequency swarm variants typical of the quasi-species were detected. Thai round eggplant samples are therefore not included in the detailed descriptions of the quasi-species populations presented below. Taken together, these results show that different variant frequency spectra may evolve, and a limited number of alternative variants may become dominant after independent infection events. Interestingly, despite the different evolutionary trajectories of quasi-species populations in independent plants, some host species shared a similar set of dominant variants that make up two-thirds of the quasi-species population: Variant1 and Variant2 in tomato and bolo maka versus Variant3 and Variant4 in chili pepper, and in Thai round eggplant.

#### 2.3.3. Phylogenetic Analysis

**Figure 2:**
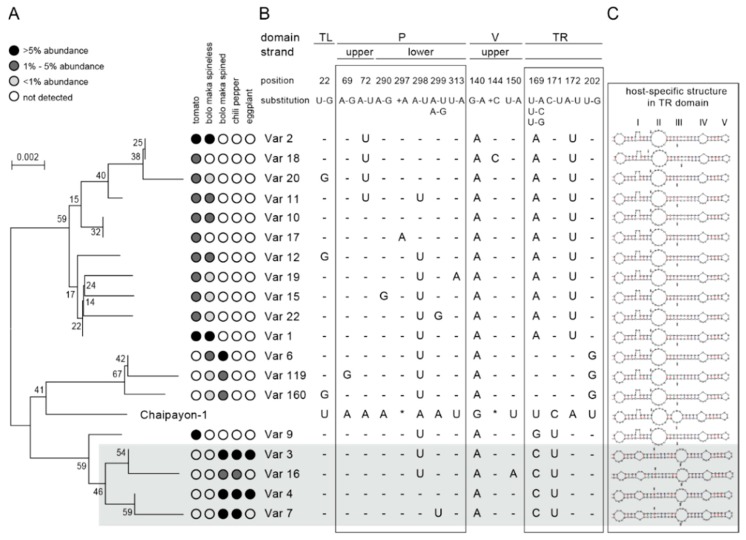
Host specific CLVd major variant analysis. (**A**) Phylogenetic tree showing the sequence similarity between major variants (>1% average relative frequency in at least one plant). Bootstrap values are indicated at the nodes. Colored circles next to the phylogenetic tree indicate abundance across plant species (black >5%; dark grey, 1–5%; light grey, 0–1%; white, 0%). (**B**) Chaipayon-1 sequence and major variant sequences at specific positions where they differ from each other. “position” indicates the position in the Chaipayon-1 reference sequence. “substitution” shows the mutation that occurred. - indicates that the sequence was the same as the Chaipayon-1 sequence. * indicates insertions. (**C**) Predicted secondary structure of each major variant. Variant3, Variant16, Variant4 and Variant7 are shaded gray because their loops are positioned at other locations compared to the other major variants.

A phylogenetic tree was created to reveal underlying sequence similarity among the 19 major variants and the CLVd Chaipayon-1 original sequence. The CLVd variants clustered into two main clades: a group composed of Variant1 and Variant2 and their respective sets of minor variants (Variant1: Variant12, Variant19, Variant15, Variant22, and Variant2: Variant17, Variant10, Variant11, Variant20, Variant18) and a group containing a sub-cluster with Variant3 and Variant4 (and closely related minor variants Variant16 and Variant7, respectively). The first clade contains only tomato and bolo maka isolates, while the second clade is composed of chili pepper and Thai round eggplant variants (Figure 2A,B). One variant from tomato (Variant9, 8.47% in Insaf plant 1) clustered in the chili pepper group.

### 2.4. Characteristics of the CLVd Progeny Variants

In order to understand how the 19 major variants (Figure 2) became dominant, i.e., preferentially and effectively replicated within the quasi-species population, we explored which sequence features may drive positive selection. Figure 2 lists the SNPs per variant in comparison to the original Chaipayon-1 reference sequence. All the CLVd progenies are similar to the original CLVd sequence and contain four to six SNPs. The 19 major variants showed 16 single nucleotide polymorphisms in 13 positions on the CLVd Chaipayon-1 genome in total, and in addition two single nucleotide insertions (Figure 2B).

#### 2.4.1. Detailed Description of the Major SNPs and Their Effect on the Secondary Structure

**Figure 3:**
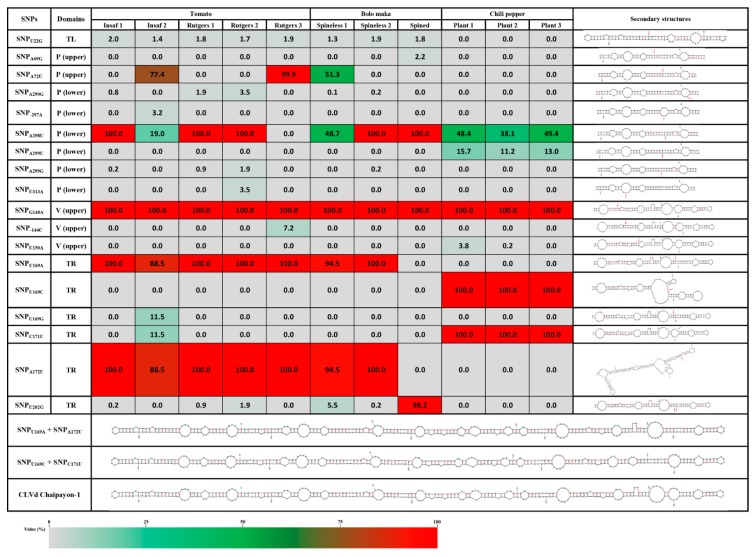
Structural consequences of major single-nucleotide polymorphisms (SNPs) of dominant variants and mutation frequency (in percentage) per individual plant. The table shows the positions and domains where mutations occurred with mutation frequency in percentage and the secondary structures. The color scale represents a mutation frequency ranging from grey (0%) over green and brown to red (100%).

The 18 major mutations occurred in most CLVd Chaipayon-1 genome domains: one SNP in the TL domain, eight SNPs and one insertion in the P domain, two SNPs and one insertion in the V domain and six SNPs in the TR domain. No SNPs were found in the C domain. In addition, all of these 18 major mutations had a different mutation frequency and their occurrence varied in each individual host plant. Furthermore, some SNPs had an effect on the viroid secondary structure (Figure 3).

##### TL Domain

The only SNP in the TL domain was found at position 22 (SNP_U22G_) and caused a loop deletion (Figure 3) in the viroid structure. This SNP, found in Variant12, Variant20 and Variant160 (Figure 2B), was not host-related and had a very low mutation frequency (Figure 3).

##### P Domain

In the P domain, eight mutations were found: SNP_A69G_ and SNP_A72U_ on the upper strand and SNP_A290G_, SNP_-297A_, SNP_A298U_, SNP_A299U_, SNP_A299G_ and SNP_U313A_ on the lower strand (Figure 3). These mutations did not affect the CLVd secondary structure except for SNP_-297A_, which added an additional loop in the stem. In addition, SNP_A72U_, SNP_A298U_ and SNP_A299U_ stabilized the secondary structure by complementary base pairing with the opposite strand. Only two SNPs, SNP_A72U_ and SNP_A298U_, showed a high mutation frequency, but they were not host specific (Figure 3). The SNP_A72U_ mutation is present in Variant2, Variant18, Variant20 and Variant11, while the SNP_A298U_ mutation occurs in all other major variants, except Variant4 and Variant7 (Figure 2B and Figure 3).

##### V Domain

Three mutations were found in the V domain: SNP_G140A_, SNP_-144C_ and SNP_U150A_. The SNP_G140A_ mutation caused complementary base-pairing and was observed in all plants with an extremely high mutation frequency of 100% (Figure 3). This mutation may have occurred at the early infection step in the first infected tomato plant (Insaf), as it is present in all major CLVd progeny variants. This suggests that the mutation is essential in the viroid infection process and was selected by the host plant–viroid interaction afterwards. SNP_-144C_ and SNP_U150A_ had a very low mutation frequency and occurred only in Variant18 and Variant16, respectively (Figure 2B and Figure 3).

##### TR Domain

In the TR domain, six major SNPs occurred: SNP_U169A_, SNP_U169C_, SNP_U169G_, SNP_C171U_, SNP_A172U_ and SNP_U202G_. Five of them are highly host specific with very high mutation frequency. SNP_U169A_ and SNP_A172U_ were present in most variants of tomato and bolo maka (except Variant6, Variant9, Variant119 and Variant160), while SNP_U169C_ and SNP_A171U_ almost exclusively occurred in the chili pepper variants (Variant3, Variant4, Variant7 and Variant16). SNP_U202G_ was only observed in Variant6, Variant119 and Variant160 that were highly prevalent in spined bolo maka (bolo maka 3) (Figure 2A, B). SNP_U169A_, SNP_U169G_ and SNP_U202G_ resulted in a deletion of the third internal loop at the TR domain (Figure 2B). Interestingly, the two dominant mutations SNP_U169C_ and SNP_A172U_, induced malformed unstable structures at the end of the TR domain (Figure 3). It is known that the rod-like secondary structure is critically important in viroid life cycles and that its destruction will cause deleterious effects. Remarkably, in the chili pepper variants, SNP_U169C_ always co-occurred with SNP_C171U_, while in the tomato and bolo maka variants, SNP_A172U_ co-occurred with SNP_U169A_. The combinations of these host specific SNPs restore the stable viroid rod-like structures (Figure 2A, B and 3). This explains why the two structure destabilizing mutations (SNP_U169C_ in chili pepper and SNP_A172U_ in tomato and bolo maka) always co-occurred with their counterparts (SNP_C171U_ and SNP_U169A_, respectively) and could be inherited by viroid progenies at very high frequencies. In addition, these SNP combinations changed the TR loop structure as well: the combination of SNP_U169C_ and SNP_C171U_ added the new small loop I and deleted the largest loop II, while the combination of SNP_A172U_ and SNP_U169A_ deleted loop III (Figure 2B).

#### 2.4.2. Secondary Structure Prediction of Major Variants

The TR domain of the secondary structure of all major variants differs from the original CLVd Chaipayon-1 structure, which contains four TR terminal loops: II, III, IV and V (Figure 2C). Secondary structure prediction clustered all major variants into two host-specific groups. The tomato/bolo maka group has lost loop III at the TR end of CLVd structure while the chili pepper group has lost loop II and carries a new loop I in comparison with the original CLVd Chaipayon-1 sequence (Figure 2B,C). As we previously described, five host specific dominant SNPs (SNP_U169A_, SNP_U169C_, SNP_C171U_, SNP_A172U_ and SNP_U202G_) effected the change in secondary structure at the TR domain which might be crucial for new host species infection.

Unlike the other tomato variants, Variant9, which was dominant in tomato plant 2 (Insaf), did not contain the SNP_A172U_–SNP_U169A_ combination. Yet, analysis of the secondary structure conformation, shows that this variant does fit in the tomato/bolo maka group. Loop II is still present, while Loop III, typical for the chili pepper isolates, is absent (Figure 2A–C).

It appears that the major CLVd variants cluster into two subgroups (tomato-bolo maka versus chili pepper). Thus, host specificity is better explained by the secondary structure of variants, than by their primary sequence.

## 3. Discussion

In this study on the evolution of CLVd quasi-species populations, we introduced the amplicon sequencing technique as an experimental tool for high-throughput variant spectrum profiling. Deep sequencing enables sensitive detection of the entire sequence spectrum of the quasi-species variant population, including rare variants.

Essentially, this methodology provides several benefits for viroid variant spectrum profiling: (1) the entire genome (368**–**374 bp) can be sequenced as a single molecule, thus excluding the need for short-read sequence assembly for variant identification; (2) short-read sequence assembly does not allow for assignment of co-occurring double mutations if these are more distant than the read length; (3) massive parallel sequencing with relatively low levels of sequence error enables discrimination of rare variants in a large population of highly similar viroid sequence variants, (4) reproducible detection of variant spectra across PCR replicates both in terms of variant identity and quantitative relative frequency estimations were demonstrated, (5) sample specific indexing makes it possible to pool hundreds of samples in a single MiSeq sequencing run and still obtain sufficient read depth (10–100k) to saturate rare variant detection. Previous high throughput sequencing methods such as RNA sequencing are limited by the read length (typically up to 2 × 150 bp when using Illumina). Using those methods, complete genome sequences cannot be explored; for example, it is not known which SNPs are combined in single CLVd variants. Although our amplicon sequencing technique involves loss of information at the primer region, the primer binding site is largely situated in the terminal left (TL) domain and has a very highly conserved sequence with around 80 nucleotides, making it highly suitable for CLVd specific primer design. Taken together, we have demonstrated the potential of whole genome deep amplicon sequencing for quantitative high-resolution temporal profiling or monitoring of quasi-species across a wide range of hosts and/or environments. This methodology thus makes it possible to capture the evolutionary dynamics of quasi-species evolution *in planta*. In turn, this makes it possible to identify the genomic regions involved in the viroid–host interaction and ultimately to reconstruct the molecular mechanism underlying host-specific quasi-species evolution.

Our discussion continues with aspects of the infection and the quasi-species evolution process based on detailed analysis of variant spectra, including infectiousness, host-specificity, (co-) occurrence of SNPs in variants and the interaction between host-specificity, SNP hotspots and RNA secondary structure stability.

### 3.1. Infectivity of Infectious dsDNA CLVd Clones

Our study has provided crucial information about the host specific infectious CLVd variants (Chaipayon-1 and Niruj-18) along with non-infectious CLVd variants (PC-2-Pa29, PC-2-Pa54, Solanum 1, Solanum 4, LPng19-4c1 and LP1-6c5) across a range of host species. The CLVd-Chaipayon-1 and Niruj isolate appeared to be tomato-specific, as the infection was successful in tomato only (but not in bolo maka, Thai round eggplant, chili pepper and bell pepper). However, the infection rate of these two variants were low (only 1/6 in tomato) (Table S1). After initial successful infection on the first tomato, all other plant species tested could be successfully inoculated by the first infected tomato plant material containing variants that were infectious on other plant hosts after mechanical transmission. It is possible that the first infection produced a swarm of CLVd progeny variants with the potential to infect solanaceous plants. However, the infection rate of the first infection assay was very low. The reason for this may be that infectivity of circular or multimeric cDNA inoculum prepared by ligating monomeric cDNA (which was not infectious by itself; data not shown) in vitro was not sufficient. It is necessary to further investigate whether they are truly infectious variants. Previous studies similarly reported non-infectious viroid variants of PSTVd, CEVd and CbVd. Several non-infectious PSTVd variants were found in tomato plants [25,43,44,45,46,47,48,49]. Similar to our case, Gora-Sochacka et al. [25] found three non-infectious PSTVd-intermediate variants (I2-50, I4-37 and I4 VI-17) among a swarm of infectious variants after inoculation with infectious cDNAs. In later work, two of these non-infectious variants (I2-50 and I4-37) showed very low infectivity with RNA transcripts inoculation [27]. In addition, they found that the infectivity of PSTVd variants depends on their sequence [25,27]. Similarly, non-infectious CEVd progenies (CEVd-PVs U30C, G128A and U182C) were found after inoculation with RNA transcripts from wild-type CEVd [31]. Similarly, infectious transcripts of CbVd-3 were able to infect *Coleus* with the “red leaf with green ruffled edge” phenotype, but not “red leaf with round edge”, “red leaf with green round edge” and “green leaf with ruffled edge” phenotypes [34].

### 3.2. Mutation Distribution in CLVd Chaipayon-1 Progeny Population in Different Host Species

Most mutations were found in the P, V and TR domain. Mutations were rare in the TL domain and were absent in the C domain. This can be explained by the occurrence of so-called “lethal mutations”, mutations that result in the death of pathogenic organism/agent. Lethal mutations have low population frequencies and cannot be passed on to the new progeny population [18]. The central conserved region (CCR) and the terminal conserved region (CUCGUGGUUCCUGUGG in TL domain) have a key function in pospiviroid replication [50]. Therefore, any mutations at these sites would be selected against.

Of all SNPs, only eight dominant SNPs with a high mutation frequency (“hotspots”), were found; two in the P domain, one in the V domain and five in the TR domain (Figure 3). However, only dominant mutations in the TR domain showed host specificity.

When studying the mutation distribution across the variant sequences, we found one main mutation (SNP_G140A_) that occurred in all variants in all host plants. This indicates that this mutation may have occurred at the very first infection step and that this SNP might be essential in the CLVd infection process. In addition, SNP_G140A_, located in the conserved motif “GACCAGUGGCG**A**GCGCCC” (SNP_G140A_ is indicated in bold) in the V domain can be found in all CLVd Asia isolates. This G to A mutation is also found in sequences from most CLVd Asia isolates deposited in GenBank, such as Accessions No. DQ923059, AM698095, JF742633, KC143289, JF446915, JF446931, JF446933, JF446937 and AM698093. This can infer that the base “A” in this motif might be important for CLVd Asia isolates in the replication and infection process, such as escaping from the host defense mechanism (RNA silencing), host trafficking or cell to cell and long-distance movement. Similarly, Podstolski et al. reported about an invariable A nucleotide at positions 173 and 310 that seems essential for all PSTVd progeny genomes for successful host infection [27]. This would explain why this mutation was selected from the very first replication round and became the most dominant SNP present in all CLVd-Chaipayon-1 progeny variants during evolution and disease development.

Five other main SNPs (SNP_U169A_, SNP_U169C_, SNP_A171U_, SNP_A172U_ and SNP_U202G_) were host specific. These mutations may have occurred after the second inoculation to the individual different host plant species (tomato, bolo maka and chili pepper), and may be needed to facilitate the introduction and adaptation of this CLVd variant to different host species. SNP_U202G_ appeared with high frequency only in spined bolo maka variants, but not in any of the other host plants (Figure 2).

The other four major SNPs were found in the conserved motif “GUARUCCC**N**R**YW**GAAACAGGGUUU” (bold letter indicates the SNPs at position 169, 171 and 172, respectively) in the TR domain, which is present in all CLVd isolates from GenBank (except Accession No. JF446927 and KC143293). Secondary structure prediction of the viroid variants revealed that the change of nucleotide on the position 169 and 171 can affect the TR terminal structure, leading to an unstable conformation that will negatively affect the selection of that viroid variant. In our work, we observed that the combination of two SNPs is needed to restore a perfect rod-like structure in most of the CLVd-Chaipayon-1 variants. This emphasizes how essential the rod-like structure is for CLVd, and by extension to other pospiviroids, in the pathogen–host interaction.

### 3.3. Host-Driven Adaptation

We found that the terminal right domain might serve as “host adaptation region” for CLVd, since two different TR structures among major variants were observed which correspond to the host plant specificity (tomato-bolo maka variants have loops I, II, IV and V, while chili pepper variants have loops I, III, IV and V). Our results lead to the hypothesis that the TR conserved motif GUARUCCC**N**R**YW**GAAACAGGGUUU might play an important role as a host adaptation region. Virp1, a bromodomain-containing protein with an atypical RNA binding domain and nuclear localization signal activity, was reported as an indispensable host factor for in-host viroid propagation. Virp1 has been shown to bind strongly to the two internal loops (IL1 and IL2) in the TR domain of pospiviroids [51,52,53] and to play an important role in nucleus-specific trafficking [54,55]. This CLVd conserved motif (nucleotides 161-184) overlaps with the two R stretches of the RY motifs, IL1 (nucleotides 155–159 as loop III) and IL2 (nucleotides 176–180 as loop IV) is critically involved in viroid propagation via binding of Virp1 [53]. It therefore seems possible that the CLVd progenies might adapt their TR internal structures to optimize binding with different host Virp1 proteins, thus providing an adaptive advantage such as enhanced fitness in host plants. In addition, the TR domain might play a key role in how pospiviroids can overcome the challenge of introduction into a new host species.

From a population of variants with randomly distributed mutations, the variants with highest adaptive advantage are selected and become the dominant variant in the quasi-species population. In addition, in accordance with Eigen’s original formulation of quasi-species theory, viroid progeny variants can accumulate and remain at the same level even under high mutation rate, as proven by the high number of dominant variants in their respective hosts [22,56,57].

## 4. Materials and Methods

### 4.1. Preparation of Infectious dsDNA CLVd Chaipayon-1

In 2014, CLVd isolate Chaipayon-1 (GenBank Accession No. KM214216) was detected in a tomato seed production facility. By using CLVd specific PC-2 primers (c-PC-2: TGTTTCWRCDGGGATTACTCCTG and h-PC-2: GGGTTTTCACCCTTCCTTTC; Figure S4) (at the position 153–197), the 367 bp full-length CLVd genome sequence was amplified [16] and was cloned into plasmid pGEM^®^-T Easy Vector and maintained in *E. coli* DH5α [8]. Recombinant plasmid containing full-length CLVd isolate Chaipayon-1 was purified from *E. coli* cell by PureYield™ Plasmid Miniprep System (Promega Corporation, Madison, WI, USA). The linear full-length CLVd dsDNA was amplified with PC-2 primers with following PCR cycles: 94 °C for 3 min, followed by 40 cycles of 40 s at 94 °C, 40 s at 56 °C and 40 s at 72 °C, and final extension at 72 °C for 10 min [58]. The 50 µL PCR reaction contained 5 µL of 10× *Pfu* Buffer, 0.2 mM of dNTPs, 2 mM MgSO_4_, 0.16 mM of each primers, 1.5 U of *Pfu* DNA polymerase with proofreading activity (Thermo Fisher Scientific Inc., Waltham, MA, USA) and 120 ng of purified recombinant plasmid. Next, the CLVd amplicon was purified with the SmartPure PCR Kit (Kaneka Eurogentec S.A., Seraing, Belgium), and concentration and purity were measured with a NanoDrop ND1000 Spectrophotometer (Isogen Life Science, De Meern, The Netherlands) and 2% agarose gel electrophoresis. The ligation reaction contained 5% (*w*/*v*) of polyethylene glycol MW 6000 and 5U of T4 DNA ligase (New England BioLabs Inc., Ipswich, MA, USA) and 10 ng/µL blunt-end linear DNA to obtain the highest chance of self-circularization [59]. The ligation reaction was incubated overnight at 16 °C and inactivated by incubation at 65°C for 10 min [60,61,62,63]. To examine the efficiency of the ligation reaction, PCR was performed with a second pair of CLVd-specific primers (c-CLVd-infect: TGCAGGGTCAGGTGTGAACCAC and h-CLVd-infect: GCCATGCAAAGRAAAAAGAAYGGG) (at the position 23–68) (Figure S4). The 20 µL PCR reaction contained 2 µL of 10× PCR Buffer, 0.2 mM dNTPs, 2 mM MgCl_2_, 0.2 mM of each primer, 1 U FastStart™ *Taq* DNA Polymerase (Sigma-Aldrich, Saint Louis, MO, USA) and 1 µL of ligated products. PCR cycles included 94 °C for 3 min, followed by 40 cycles of 40 s at 94 °C, 40 s at 59 °C and 40 s at 72 °C, with final extension at 72 °C for 10 min, in a T100™ Thermal Cycler (Bio-Rad, Hercules, Ca, USA). The PCR products were analyzed using 2% agarose gel electrophoresis.

### 4.2. Plant Inoculation and Maintenance

Two-week old tomato (cv. Insaf) and three-week old bolo maka (*Solanum stramoniifolium*; both spined type and spineless type), Thai round eggplant (*Solanum melongena*) and chili pepper (*Capsicum annuum*) plants were injected with infectious dsDNA CLVd 5–10 µL (5–6 replicate plants per host) using needle and syringe. All inoculated plants were kept in a greenhouse at 27 °C. Only one tomato cv. Insaf plant showed the viroid-specific symptoms and presence of the viroid was confirmed by RT-PCR. The CLVd-infected tomato plant was used as inoculum for the second round inoculation (tomato cvs. Insaf and Rutgers, bolo maka, Thai round eggplant and chili pepper). The CLVd-positive plant was mechanically transmitted to all the new healthy plants (two-week-old tomato plants and three-week-old plants for the others) by mechanical inoculation, after which all the plants were kept under the same conditions. Leaf samples were taken after inoculated plants showed strong and specific symptoms and infection was confirmed using RT-PCR (four to eight weeks after inoculation, depending on the plant species).

### 4.3. RNA Extraction, Library Preparation and Amplicon Sequencing

Total RNA was extracted from 100 mg fresh weight CLVd-infected leaves using the Spectrum™ Plant Total RNA Kit (Sigma-Aldrich, Saint Louis, MO, USA) following the manufacturer’s protocol. Total RNA (50 µg) was subjected to reverse transcription using the iScript™ cDNA Synthesis kit (Bio-Rad, Hercules, CA, USA). c-CLVd-infect and h-CLVd-infect primers (see above), were extended with variable length indices on the 5’-end of each primer, so that each sample could be identified by a unique dual index during sample demultiplexing. PCR amplification was performed in a 20 µL reaction mixture consisting of 10 µL of BIO-X-ACT Short Mix, 0.2 mM of indexed CLVd primers and 1 µL of cDNA. PCR cycles included initiation at 94 °C for 3 min followed by 40 cycles of 40 s at 94 °C, 40 s at 59 °C and 40 s at 72 °C, and final extension at 72 °C for 10 min. For each cDNA sample, six replicate PCR amplifications were performed in parallel with separate indices. All PCR products were individually purified with Sera-Mag™ SpeedBead Carboxylate-Modified Magnetic Particles (Thermo Fisher Scientific Inc., Waltham, MA, USA), and quantified with QuantiFluor^®^ dsDNA System (Promega Corporation, Madison, WI, USA). In total, 80 ng DNA per PCR replicate were pooled and samples were split over three parallel pools. The different amplicon pools were ligated to Illumina adapters by the sequencing provider (Admera Health, South Plainfield, NJ, USA) using the KAPA Hyper Prep PCR-free ligation kit (F. Hoffmann-La Roche AG, Basel, Switzerland). All ligated libraries were sequenced on two runs of a MiSeq v3 instrument (2 × 300 bp) (Illumina, San Diego, CA, USA).

### 4.4. Read Processing and Downstream Analysis

Each ligated library was demultiplexed by a custom script using the tool sabre v1.000 (https://github.com/najoshi/sabre) to handle variable length sample indices. A read pair was only assigned to a certain sample if both its forward and reverse barcode matched without errors. After the demultiplexing, the forward and reverse reads were merged using PEAR v0.9.11 [64] with a minimum overlap of 20 bp and a final fragment length of minimum 150 and maximum 400 bp. Subsequently, the primers were removed both from the 5’ and the 3’ end using cutadapt v1.15 [65], allowing for maximum 15% errors in the primer sequence. Only fragments with both primers were retained. Next, low quality reads with a maximum expected error of 0.25 were removed using vsearch v2.7.1 (-fastq_filter) [66]. The length distribution of the remaining amplicons was checked, and an extra length filtering was done using cutadapt, keeping only fragments between 300 and 350 bp. All sequences were then reverse complemented using “fastx_reverse_complement” from the FASTX toolkit v.0.0.14 (http://hannonlab.cshl.edu/fastx_toolkit/index.html). The sequences were renamed and concatenated into a single file for further processing using the OBITools software suite v1.2.13 [67]. Dereplicated sequences ("obiuniq") with a read count of minimum 1000 over all samples were kept for further analysis ("obigrep"). Finally, the sequences were sorted by abundance ("obisort") and written to output in table format ("obitab") summarizing the counts of each amplicon variant for each sample. The resulting tables were read into R v.3.6.1 [68] for further processing and visualization using the phyloseq package [69]. The sequences from all variants and Chaipayon-1 isolate were aligned by MUSCLE (MEGA7) with default parameters [70]. The phylogenetic tree was created in MEGA7 using Neighbor-Joining (bootstrap 1000 replicates). The RNA Folding Form program (http://unafold.rna.albany.edu/?q=mfold/RNA-Folding-Form) was used to predict the thermodynamic secondary structure (at the lowest Gibbs free energy, ΔG) of all major CLVd variants and SNPs. The raw reads are available through NCBI’s Sequence Read Archive (SRA) under BioProject number PRJNA600376, and all scripts are available at Zenodo under doi 10.5281/zenodo.3701202.

## 5. Conclusions

In this work, the existence of quasi-species of CLVd populations in several host plants was demonstrated. The relationship between variant sequences, SNPs, viroid secondary structures and host species was clarified and discussed. We have found that the terminal right domains may serve as “host adaptation region” for CLVd. In addition, the fitness and equilibrium of CLVd quasi-species were illustrated here. Here, we demonstrate the power of high-throughput deep amplicon sequencing to analyze the complete viroid variant sequences, while maintaining relative frequencies of variants in the viroid quasi-species population. By directly analyzing the CLVd quasi-species population, based on the obtained nearly full-length genomes of viroid populations in different host plants, the host-driven *in planta* evolution of a specific founder sequence could be assessed.

## Supplementary Materials

Supplementary materials can be found at https://www.mdpi.com/1422-0067/21/9/3262/s1.
