# Supplementary material for: Whole-Genome Deep Sequencing Reveals Host-Driven in-planta Evolution of Columnea Latent Viroid (CLVd) Quasi-Species Populations
Source: Int J Mol Sci. 2020 May 5;21(9):3262. doi: 10.3390/ijms21093262 (PMC7246631; doi:10.3390/ijms21093262)
Supplement: Supplementary file 1 [file ijms-21-03262-s001.zip › Figure S1 Infectious dsDNA CLVd products.pdf]

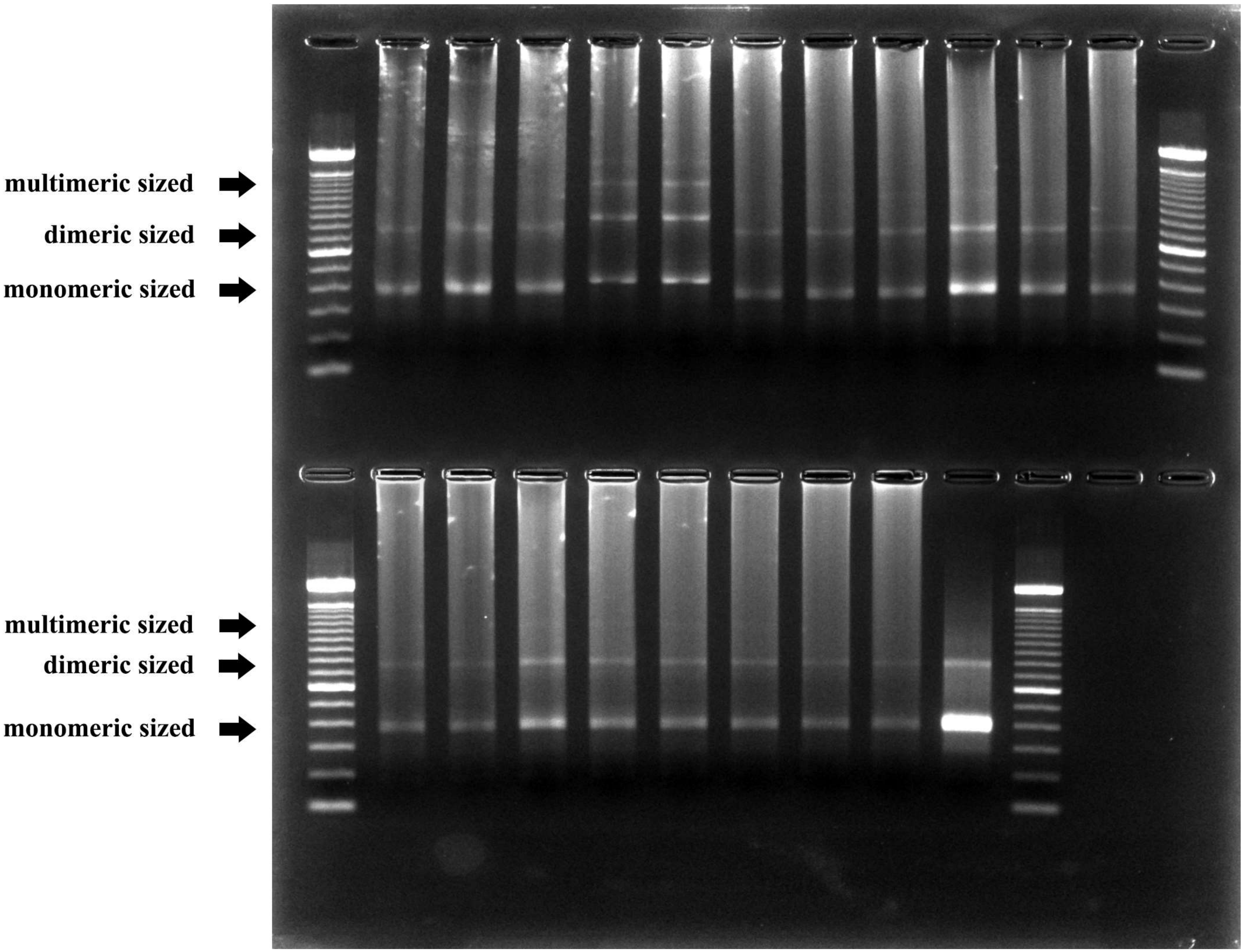
multimeric sized
dimeric sized
monomeric sized
multimeric sized
dimeric sized
monomeric sized
