# Supplementary material for: Whole-Genome Deep Sequencing Reveals Host-Driven in-planta Evolution of Columnea Latent Viroid (CLVd) Quasi-Species Populations
Source: Int J Mol Sci. 2020 May 5;21(9):3262. doi: 10.3390/ijms21093262 (PMC7246631; doi:10.3390/ijms21093262)
Supplement: Supplementary file 1 [file ijms-21-03262-s001.zip › Table S1 Summary CLVd infection experiments.pdf]

			Host plants (3-10 plants/experiment)							
**Experiments**	**CLVd isolates**	**AC numbers**	**tomato cv. Insaf F1**	**tomato cv. Rutgers**	**tomato cv. Seeda 50**	**tomato cv. Cherry**	**bolo maka**	**hot pepper**	**Thai round eggplant**	**bell pepper**
**1 (20-Sep-2017)**	PC-2-Pa29	DQ061193	0/6	na.	na.	na.	0/5	0/6	0/5	0/3
(injection)	PC-2-Pa54	DQ061192	0/6	na.	na.	na.	0/5	0/6	0/5	0/3
	Solanum 1	JF742632	0/6	na.	na.	na.	0/5	0/6	0/5	0/3
	Solanum 4	JF742633	0/6	na.	na.	na.	0/5	0/6	0/5	0/3
	Chaipayon-1	KM214216	**1/6**	na.	na.	na.	0/5	0/6	0/5	0/3
	Niruj-18	KM214222	**1/6**	na.	na.	na.	0/5	0/6	0/5	0/3
	LPng19-4c1	JF446934	0/6	na.	na.	na.	0/5	0/6	0/5	0/3
	LP1-6c5	JF446916	0/6	na.	na.	na.	0/5	0/6	0/5	0/3
**2 (4-Feb-2019)**	PC-2-Pa29	DQ061193	na.	0/5	0/5	0/3	0/5	0/3	0/3	na.
(injection)	Solanum 1	JF742632	na.	0/5	0/5	0/3	0/5	0/3	0/3	na.
	Chaipayon-1	KM214216	na.	0/5	0/5	0/3	0/5	0/3	0/3	na.
	LPng19-4c1	JF446934	na.	0/5	0/5	0/3	0/5	0/3	0/3	na.
**3 (16-Apr-2019)**	PC-2-Pa29	DQ061193	na.	0/10	0/5	na.	0/5	na.	na.	na.
(injection)	Solanum 1	JF742632	na.	**1/10**	0/5	na.	0/5	na.	na.	na.
	Chaipayon-1	KM214216	na.	0/10	0/5	na.	0/5	na.	na.	na.
(grafting)	PC-2-Pa29	DQ061193	na.	na.	na.	na.	0/5	na.	na.	na.
	Solanum 1	JF742632	na.	na.	na.	na.	0/5	na.	na.	na.
	Chaipayon-1	KM214216	na.	na.	na.	na.	0/5	na.	na.	na.

All the plants were inoculated directly by dsDNA CLVds
All the infection results were tested by RT-PCR
