# Supplementary figures and images for: Whole-Genome Deep Sequencing Reveals Host-Driven in-planta Evolution of Columnea Latent Viroid (CLVd) Quasi-Species Populations
Source: Int J Mol Sci. 2020 May 5;21(9):3262. doi: 10.3390/ijms21093262 (PMC7246631; doi:10.3390/ijms21093262)

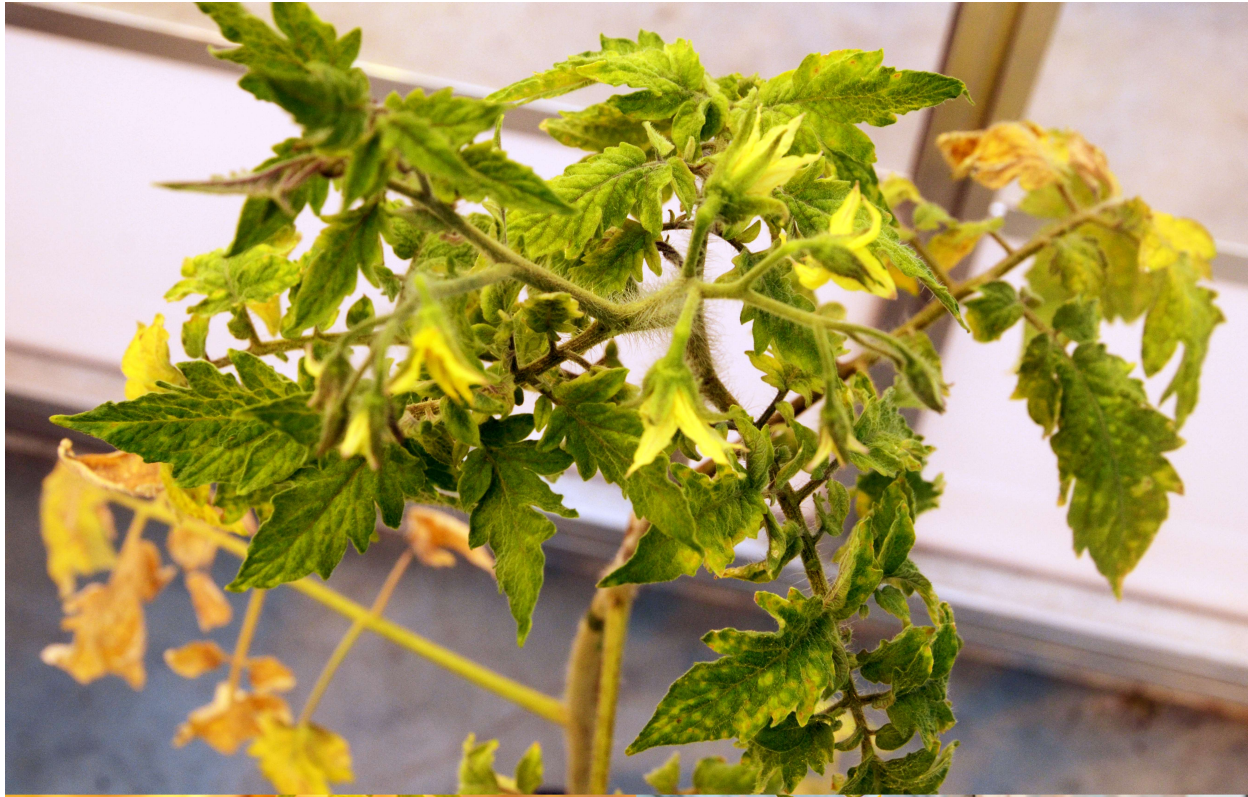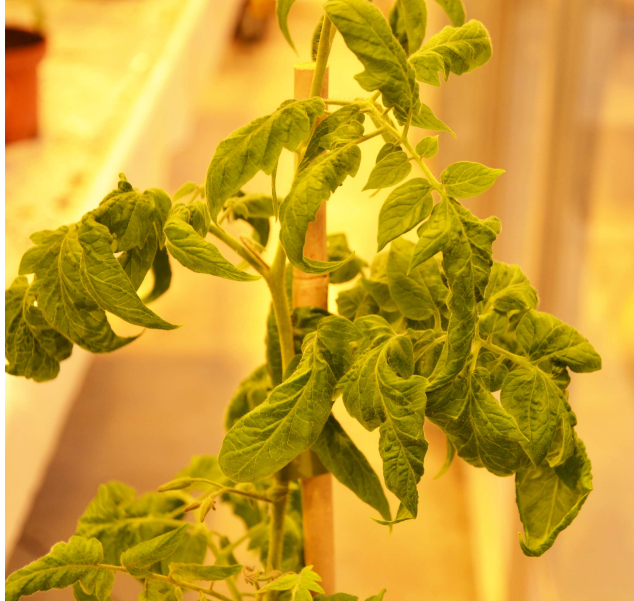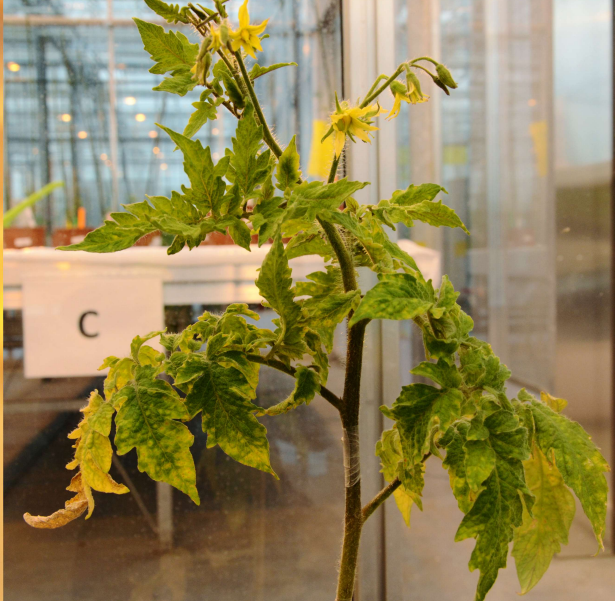

Supplement: Supplementary file 1 [file ijms-21-03262-s001.zip › Figure S2 CLVd-Chaipayon-1 symptoms on tomato Insaf F1.pdf]

A

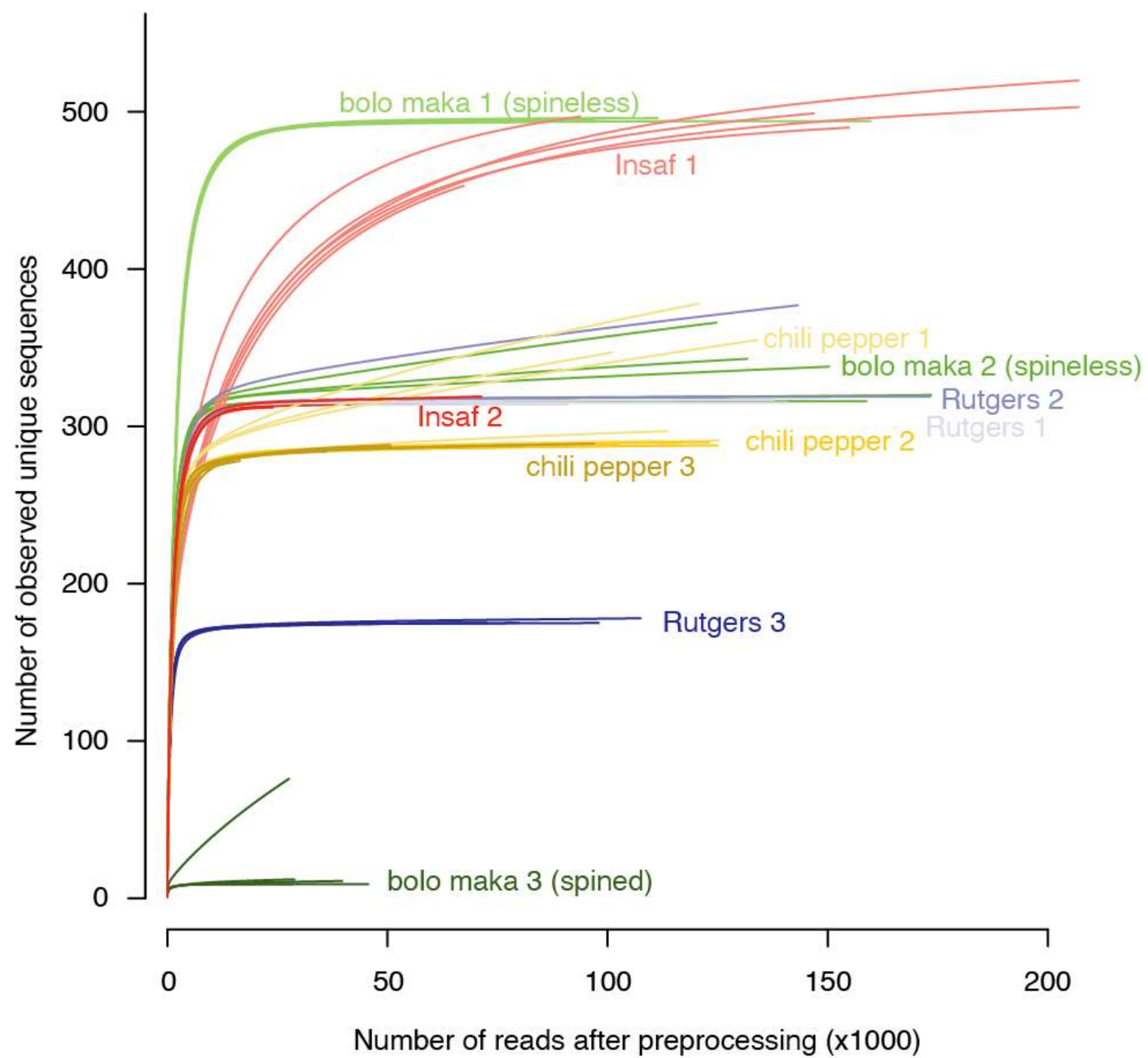

Supplement: Supplementary file 1 [file ijms-21-03262-s001.zip › Figure S3 Rare fraction plot.pdf]

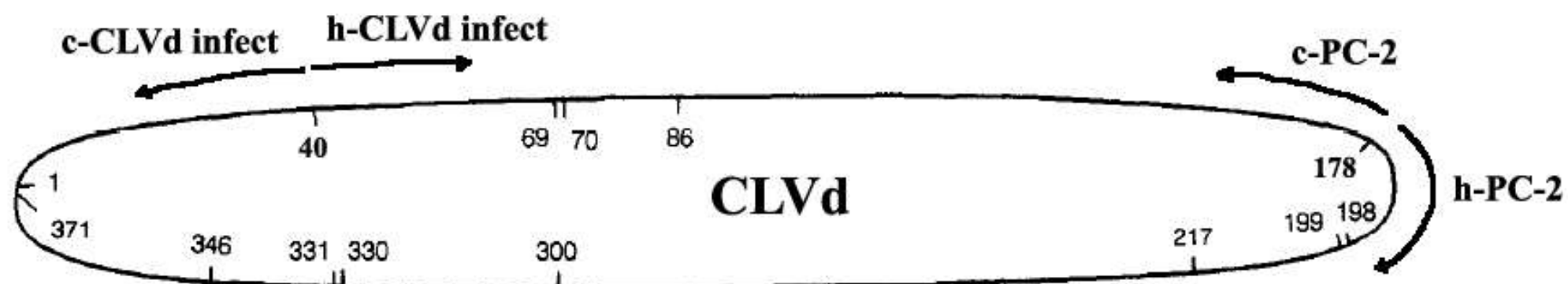

Supplement: Supplementary file 1 [file ijms-21-03262-s001.zip › Figure S4 Scheme of the CLVd primers.pdf]
